# Tremor-associated short tandem repeat intermediate and pathogenic expansions in familial essential tremor

**DOI:** 10.1093/braincomms/fcae217

**Published:** 2024-06-29

**Authors:** Xun Zhou, Runcheng He, Sheng Zeng, Mingqiang Li, Hongxu Pan, Yuwen Zhao, Zhenhua Liu, Qian Xu, Jifeng Guo, Xinxiang Yan, Jinchen Li, Beisha Tang, Qiying Sun

**Affiliations:** Department of Geriatric Neurology, Xiangya Hospital, Central South University, Changsha, Hunan 410008, China; Department of Neurology, Xiangya Hospital, Central South University, Changsha, Hunan 410008, China; Department of Neurology, Xiangya Hospital, Central South University, Changsha, Hunan 410008, China; Department of Geriatrics, The Second Xiangya Hospital, Central South University, Changsha, Hunan 410011, China; Department of Neurology, Multi-Omics Research Center for Brain Disorders, The First Affiliated Hospital, Hengyang Medical School, University of South China, Hengyang, Hunan 421000, China; Department of Neurology, Xiangya Hospital, Central South University, Changsha, Hunan 410008, China; Department of Neurology, Xiangya Hospital, Central South University, Changsha, Hunan 410008, China; Department of Neurology, Xiangya Hospital, Central South University, Changsha, Hunan 410008, China; National Clinical Research Center for Geriatric Disorders, Xiangya Hospital, Central South University, Changsha, Hunan 410008, China; Key Laboratory of Hunan Province in Neurodegenerative Disorders, Central South University, Changsha, Hunan 410008, China; Department of Neurology, Xiangya Hospital, Central South University, Changsha, Hunan 410008, China; National Clinical Research Center for Geriatric Disorders, Xiangya Hospital, Central South University, Changsha, Hunan 410008, China; Key Laboratory of Hunan Province in Neurodegenerative Disorders, Central South University, Changsha, Hunan 410008, China; Department of Neurology, Xiangya Hospital, Central South University, Changsha, Hunan 410008, China; National Clinical Research Center for Geriatric Disorders, Xiangya Hospital, Central South University, Changsha, Hunan 410008, China; Key Laboratory of Hunan Province in Neurodegenerative Disorders, Central South University, Changsha, Hunan 410008, China; Department of Neurology, Xiangya Hospital, Central South University, Changsha, Hunan 410008, China; National Clinical Research Center for Geriatric Disorders, Xiangya Hospital, Central South University, Changsha, Hunan 410008, China; Department of Geriatric Neurology, Xiangya Hospital, Central South University, Changsha, Hunan 410008, China; National Clinical Research Center for Geriatric Disorders, Xiangya Hospital, Central South University, Changsha, Hunan 410008, China; Center for Medical Genetics, School of Life Sciences, Central South University, Changsha, Hunan 410008, China; Department of Neurology, Xiangya Hospital, Central South University, Changsha, Hunan 410008, China; Department of Neurology, Multi-Omics Research Center for Brain Disorders, The First Affiliated Hospital, Hengyang Medical School, University of South China, Hengyang, Hunan 421000, China; National Clinical Research Center for Geriatric Disorders, Xiangya Hospital, Central South University, Changsha, Hunan 410008, China; Key Laboratory of Hunan Province in Neurodegenerative Disorders, Central South University, Changsha, Hunan 410008, China; Center for Medical Genetics, School of Life Sciences, Central South University, Changsha, Hunan 410008, China; Department of Geriatric Neurology, Xiangya Hospital, Central South University, Changsha, Hunan 410008, China; National Clinical Research Center for Geriatric Disorders, Xiangya Hospital, Central South University, Changsha, Hunan 410008, China; Key Laboratory of Hunan Province in Neurodegenerative Disorders, Central South University, Changsha, Hunan 410008, China

**Keywords:** essential tremor, short tandem repeat, intermediate repeats, pathogenic expansion

## Abstract

There is an obvious clinical–pathological overlap between essential tremor and some known tremor-associated short tandem repeat expansion disorders. The aim is to analyse whether these short tandem repeat genes, including *ATXN1*, *ATXN2*, *ATXN3*, *CACNA1A*, *ATXN7*, *ATXN8OS*, *ATXN10*, *PPP2R2B*, *TBP*, *BEAN1*, *NOP56*, *DAB1*, *ATN1*, *SADM12* and *FMR1*, are associated with familial essential tremor patients. Genetic analysis of repeat sizes in tremor-associated short tandem repeat expansions was performed in a large cohort of 515 familial essential tremor probands and 300 controls. The demographic and clinical features among carriers of pathogenic expansions, intermediate repeats and non-carriers were compared. A total of 18 out of 515 (18/515, 3.7%) patients were found to have repeats expansions, including 12 cases (12/515, 2.5%) with intermediate repeat expansions (one *ATXN1*, eight *TBP*, two *FMR1*, one *ATN1*), and six cases (6/515, 1.2%) with pathogenic expansions (one *ATXN1*, one *ATXN2*, one *ATXN8OS*, one *PPP2R2B*, one *FMR1*, one *SAMD12*). There were no statistically significant differences in intermediate repeats compared to healthy controls. Furthermore, there were no significant differences in demographics and clinical features among individuals with pathogenic expansions, intermediate repeat expansions carriers and non-carriers. Our study indicates that the intermediate repeat expansion in tremor-associated short tandem repeat expansions does not pose an increased risk for essential tremor, and rare pathogenic expansion carriers have been found in the familial essential tremor cohort. The diagnosis of essential tremor based solely on clinical symptoms remains a challenge in distinguishing it from known short tandem repeat expansions diseases with overlapping clinical–pathological features.

## Teaser

In the study of 515 familial essential tremor patients, Zhou *et al*. reported that 3.7% of patients carried intermediate expansion and 1.2% had pathogenic expansion of tremor-associated short tandem repeats. This work underscores the importance of considering these mutations in these patients, highlighting the diagnostic complexities associated with the condition.

## Graphical Abstract

**Graphical Abstract:**
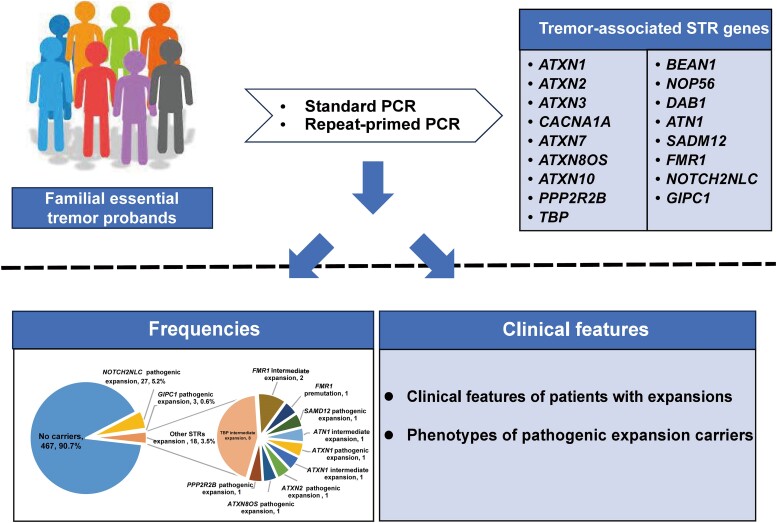

## Introduction

Short tandem repeats (STRs) are abundant nucleotide repeat sequences dispersed throughout the human genome, typically consisting of 2–6 nucleotides in length that are repeated consecutively.^1,2^ These repetitive sequences are prone to variations in the number of repeats during DNA replication. Expanded STRs can result in pathogenic effects, and more than 50 disorders are known to be associated with expanded STR loci. These STRs are generally categorized based on their repeat lengths into three groups: normal, intermediate (characterized by a repeat size below the pathogenic length but exhibiting genetic instability, with the potential for expansion in further generations), premutation [with 55–200 GGC repeats of *FMR1* gene, carrying an increased risk for fragile X tremor/ataxia syndrome (FXTAS)] and pathogenic expansions. Tremors are observed in nearly one-third of these disorders, with many involving cerebellar changes, including spinocerebellar ataxias (SCAs), Dentatorubral-pallidoluysian atrophy (DRPLA), FXTAS and familial cortical myoclonic tremor and epilepsy (FCMTE), *NOTCH2NLC* related repeat expansion disorders and *GIPC1* related movement disorders.^3-6^

Essential tremor (ET) is one of the most common tremor disorders, characterized by a 4–12 Hz action tremor primarily affecting the upper limbs, usually occurring during various voluntary movements.^7,8^ The tremors may also extend to the lower limbs, head, voice, lip and other body regions. Recent clinical and pathological investigations have increasingly supported the notion of cerebellar involvement in ET. Firstly, ∼28–40% of ET patients exhibit intention tremors in their arms.^9,10^ Recent studies have also revealed abnormalities in tandem gait and oculomotor deficits in ET patients^11-13,14,15^ providing strong indicators of cerebellar dysfunction. Intention tremor is caused by disturbances of the cerebellar outflow projection system mediated by the dentate nucleus and superior cerebellar peduncle.^16^ Tandem gait and oculomotor impairment represent symptoms of cerebellar regulation of the midline and caudal vermis, respectively.^11,17^ Secondly, recent structural and functional image studies have shown a correlation of grey matter decreases in cerebellum with ET, and disturb in cerebello-dentato-thalamic connectively in ET.^16,18,19^ Thirdly, degenerative changes primarily affect the cerebellum in ET, particularly affecting the Purkinje cells (PCs) and adjacent neuronal populations.^20,21^ Furthermore, genetic anticipation, a characteristic feature of repeat expansion diseases, has been also observed in some ET families.^22,23^ Therefore, there is an obvious clinical–pathological overlap between ET and these tremor-associated STR expansion disorders.

Prior investigations have reported significant familial aggregation in ET, with twin studies suggesting high heritability (45–90%).^24-26^ Despite the identification of up to 50 genes/loci, the lack of consistent replication suggests a high degree of genetic heterogeneity.^27^ Recent studies have revealed an association between ET and repeat expansions in *NOTCH2NLC*, with ∼5% of familial ET patients carrying pathogenic expansions.^5,28^ Given the evident overlap in clinical and pathological characteristics between ET and these tremor-associated STR expansion disorders, familial ET patients may also carry these STR expansions.

In this study, we investigated a cohort of 515 familial ET cases and 300 healthy controls with tremor-associated STR genes: *ATXN1* (SCA1), *ATXN2* (SCA2), *ATXN3* (SCA3), *CACNA1A* (SCA6), *ATXN7* (SCA7), *ATXN8OS* (SCA8), *ATXN10* (SCA10), *PPP2R2B* (SCA12), *TBP* (SCA17), *BEAN1* (SCA31), *NOP56* (SCA36), *DAB1* (SCA37), *ATN1* (DRPLA), *SADM12* (FAME1) and *FMR1* (FXS/FXTAS), to determine whether familial ET are associated with these genes expansions, and assess the impact of expansions on these patients.

## Methods

### Subjects

**Figure 1:**
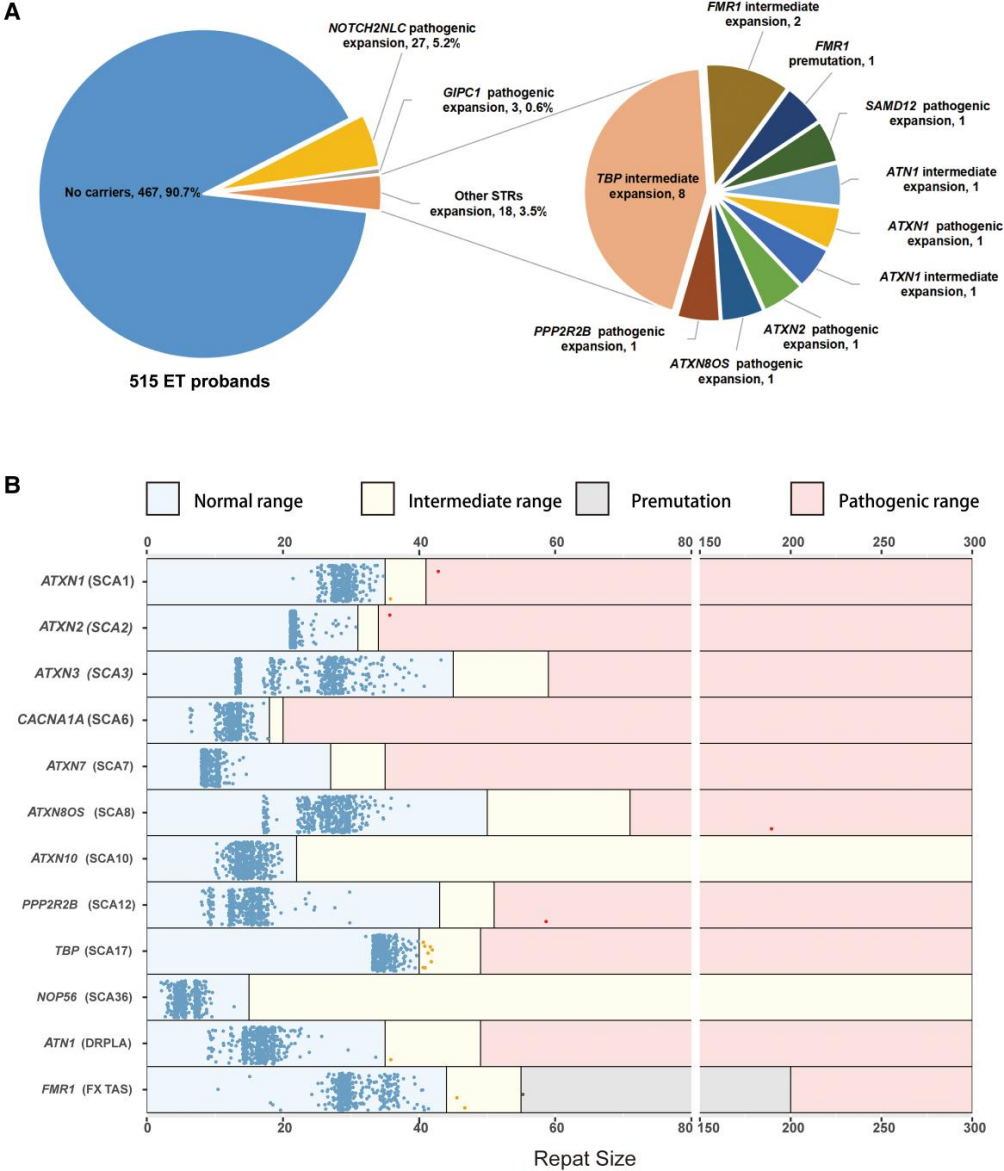
**The screening results of tremor-associated STRs.** (**A**) The frequencies of known tremor-associated STRs expansions in 515 familial ET probands. (**B**) Results of known STRs size detection in 515 familial ET probands [*BEAN1* (SCA31), *DAB1* (SCA37) and *SAMD12* (FCMTE1) are not shown since the disease is caused by alternative pathogenic repeat insertions]. STRs, short tandem repeats; SCA, spinocerebellar ataxia; DRPLA, Dentatorubral-pallidoluysian atrophy; FXTAS, fragile X tremor/ataxia syndrome; FMCTE, familial cortical myoclonic tremor and epilepsy.

We conducted a retrospective review of medical records and clinical assessment data of ET patients recruited between 2013 and 2022 at the National Clinical Research Center for Geriatric Disorders, Xiangya Hospital, Central South University. A total of 515 probands with ET, with a mean age of 53.8 ± 16.1 and 45.6% males, meeting the guidelines of the Consensus Statement of the Movement Disorders Society (2018),^29^ underwent successful screening for the tremor-associated STR genes. In our previous study, we screened for *NOTCH2NLC* and *GIPC1* GGC repeat expansions in this cohort, identifying 27 (5.2%) probands with *NOTCH2NLC* repeat expansions and 3 (0.6%) probands with *GIPC1* repeat expansions^28,30^ (Fig. 1A).

Comprehensive clinical assessments were conducted, including the collection of family history, demographic characteristics, clinical data and neurological examination. The severity of ET was evaluated using the Tremor Research Group Essential Tremor Rating Assessment Scale (TETRAS). Non-motor symptoms were assessed using the Non-Motor Symptoms Scale (NMSS), cognitive function was evaluated with the Mini-Mental State Examination (MMSE), and cognitive impairment was defined based on the following criteria: illiterate (≤17), elementary education (20) and middle school or higher education (≤24). Dystonia was assessed using the Unified Dystonia Rating Scale, and a score ≥ 1 was defined as questionable dystonia posturing. Tandem gait (10 steps) was evaluated by straight line walk, and at least two missteps out of 10-step trials were defined as impaired tandem gait.

A control group comprising 300 individuals without any neurological diseases was recruited from the medical examination of Xiangya Hospital, matched on age, gender and ethnicity (Mongolian).^31^ After obtaining written informed consent, peripheral blood samples were collected from all participants. Genomic DNA was extracted from peripheral blood via a standardized phenol-chloroform method. This study was approved by the Ethics Committee of Xiangya Hospital of Central South University.

### Genotyping for tremor-associated STRs

The repeats genotyping of tremor-associated STRs were determined by polymerase chain reaction (PCR) using fluorescently labelled primers respectively (primers and PCR conditions are available upon request), followed by capillary electrophoresis on an ABI3730xl genetic analyser. Data were analysed using GeneMarker software v2.6, and allele sizes were defined based on comparison with the molecular weight standard Gene Scan GS550 (Applied Biosystems, Inc.). The range threshold of expanded repeats in tremor-associated STRs is provided in Supplementary Table 1.

### Statistical analysis

The chi-squared and Fisher's exact tests were used to compare the frequency of repeat lengths between cases and controls. To analyse the influence of expanded STRs on the clinical features, Mann–Whitney U test was performed on continuous variables between two groups comparison, and Kruskal–Wallis test was used in three groups. The Bonferroni test was used in multiple-comparison correction. The statistical analysis was performed by SPSS (version 26.0) and R Statistical Software (version 4.2.2, R package ggplot, geom_bar and geom_jitter).

## Results

### Frequencies of intermediate and pathologic STR expansions in ET

We successfully screened the repeats of tremor-associated STRs in 515 probands with ET and 300 control individuals. Within the cohort of 515 familial ET probands, we identified repeat expansions in 18 cases (18/515, 3.5%). Of these, 12 cases (12/515, 2.3%) displayed intermediate repeats (one *ATXN1*, eight *TBP*, two *FMR1* and one *ATN1*), and six cases (6/515, 1.2%) exhibited pathogenic (premutation) expansions (one *ATXN1*, one *ATXN2*, one *ATXN8OS*, one *PPP2R2B*, one *FMR1* and one *SAMD12*) (Fig. 1A). Among the 300 healthy controls, intermediate repeats were observed in four individuals (1.3%) (three *SCA17* and one *FMR1*).^31^ Notably, no significant differences in the frequency of intermediate or pathogenic repeats were observed between ET probands and controls (Table 1). Furthermore, all expanded repeat alleles detected in individuals with ET and healthy controls were found to be heterozygotes. The sizes of STRs detected by PCR are illustrated in Fig. 1B.

**Table 1:** Genotyping results for repeat alleles in tremor-associated STRs in ET group and healthy controls

	Normal alleles	Intermediate alleles	Pathogenic alleles	Normal alleles	Intermediate alleles	Pathogenic alleles	*P* value (intermediate alleles)	*P* value (pathogenic alleles)
*ATXN1* (SCA1)	968	1	1	600	0	0	1.000	1.000
*ATXN2* (SCA2)	969	0	1	600	0	0		1.000
*ATXN3* (SCA3)	970	0	0	600	0	0		
*CACNA1A* (SCA6)	970	0	0	600	0	0		
*ATXN7* (SCA7)	970	0	0	600	0	0		
*ATXN8OS* (SCA8)	969	0	1	600	0	0		1.000
*ATXN10* (SCA10)	970	0	0	600	0	0		
*PPP2R2B* (SCA12)	969	0	1	600	0	0		1.000
*TBP* (SCA17)	970	8	0	597	3	0	0.671	
*BEAN1* (SCA31)	970	0	0	600	0	0		
*NOP56* (SCA36)	970	0	0	600	0	0		
*DAB1* (SCA37)	970	0	0	600	0	0		
*ATN1* (DRPLA)	969	1	0	600	0	0	1.000	
*FMR1* (FXTAS)	967	2	1^a^	599	1	0	1.000	1.000
*SAMD12* （FCMTE）	969	0	1	600	0	0		1.000

^a^Premutation alleles.

### Clinical features of patients with expansions

The mean ages at onset were 36.0, 47.3 and 41.6 for individuals with pathogenic expansions, intermediate repeat expansions and non-carriers, respectively. While patients with pathogenic expansions had the earliest age at onset, no significant differences were observed among the three groups, and there were no significant correlations between the sizes of these STRs and age at onset (Supplementary Fig. 1). Notably, there were also no significant differences in the distribution, type or severity of tremors, non-motor symptoms or the presence of ET-plus across the three groups (Table 2). We further conducted a comparative analysis, contrasting carriers of *SCAs* expansions (10 cases with intermediate expansion and 4 with pathogenic expansion) and *FMR1* expansions (2 cases with intermediate expansion and 1 with pathogenic expansion) with non-carriers. *SAMD12* expansion was excluded from statistical analysis due to a limited number of cases. The comparison did not reveal any significant differences between expansion carriers and non-carriers of the respective expansions (Supplementary Table 2).

**Table 2:** Demographic and clinical features in carriers of pathogenic repeats, intermediate alleles and non-carriers

	Carriers of pathogenic expansions, *n* = 6	Carriers of IAs, *n* = 12	Non-carriers, *n* = 467	*P* (all)	Adj. *P* (pathogenic repeats versus IAs)	Adj. *P* (pathogenic repeats versus non-carriers)	Adj. *P* (IAs versus non-carriers)
Sex, male, *n* (%)	3 (50.0)	7 (58.3)	254 (54.4)	1.000	1.000	1.000	1.000
Age, years, mean (SD)	51.0 (10.1)	56.0 (17.3)	53.8 (16.1)	0.643	0.250	0.454	0.592
Duration (SD)	16.2 (15.2)	9.2 (4.9)	11.9 (9.6)	0.711	0.521	0.632	0.497
Education, years, mean (SD)	10.6 (2.9)	13.0 (5.2)	10.2 (4.0)	0.383	0.284	0.953	0.167
Head tremor, *n* (%)	2 (33.3)	2 (16.7)	153 (32.8)	0.510	0.569	1.000	0.387
Face tremor, *n* (%)	1 (16.7)	2 (16.7)	122 (26.1)	0.829	1.000	1.000	0.686
Voice tremor, *n* (%)	3 (50.0)	2 (16.7)	131 (28.1)	0.368	0.268	0.360	0.577
Upper limbs tremor, *n* (%)	6 (100.0）	12 (100.0)	467 (100.0)	1.000	NA	NA	NA
Lower limbs tremor, *n* (%)	3 (50.0)	3 (25.0)	134 (28.7)	0.485	0.344	0.363	1.000
Postural tremor, *n* (%)	6 (100.0）	12 (100.0)	467 (97.9)	NA	NA	NA	NA
Kinetic tremor, *n* (%)	6 (100.0）	12 (100.0)	453 (97.0)	1.000	NA	1.000	1.000
Intention tremor, *n* (%)	4 (66.7)	5 (41.7)	178 (38.1)	0.363	0.620	0.210	1.000
TETRAS-I (SD)	16.3 (11.4)	9.6 (7.4)	13.4 (9.7)	0.449	0.228	0.481	0.297
TETRAS-II (SD)	19.0 (9.7)	18.0 (5.3)	17.4 (7.8)	0.872	0.573	0.660	0.776
MMSE (SD)	27.7 (2.1)	28.8 (1.2)	27.7 (2.8)	0.592	0.310	0.635	0.372
NMSS (SD)	5.2 (5.3)	8.3 (9.9)	12.1 (15.5)	0.534	0.818	0.345	0.533
ET-plus, *n* (%)	1 (16.7)	5 (41.7)	234 (50.1)	0.481	0.600	0.216	0.564
Dystonia, *n* (%)	0 (0.0)	1 (8.3)	37 (7.9)	1.000	1.000	1.000	1.000
MCI, *n* (%)	0 (0.0)	2 (16.7)	107 (22.9)	0.536	0.529	0.345	0.872
Tandem repeat, *n* (%)	0 (0.0)	1 (8.3)	54 (11.6)	1.000	1.000	0.811	1.000
Rest tremor, *n* (%)	1 (16.7)	1 (8.3)	57 (12.2)	0.840	1.000	1.000	1.000

Adj. *P* = adjusted *P* (statistically significant cut-off of *P* < 0.017 by Bonferroni correction).

IA, intermediate allele; SD, standard deviation; ET, essential tremor; TETRAS, Tremor Research Group Essential Tremor Rating Assessment Scale; MMSE, Mini-Mental State Examination; NMSS, Non-Motor Symptoms Scale; MCI, mild cognitive impairment; NA, data not available.

Our previous investigations have identified 27 familial ET patients with *NOTCH2NLC* pathogenic expansions and 3 with *GIPC1* pathogenic expansions, all lacking intermediate repeats in the two genes.^6,28^ Herein, we compared carriers of tremor-associated pathogenic expansions with those of *NOTCH2NLC* and *GIPC1* expansions. Significant differences were observed in TETRAS-I and NMSS scores, as well as the prevalence of ET-plus among the three groups (*P* < 0.05) (Table 3). Further pairwise comparisons revealed that carriers of *NOTCH2NLC* pathogenic expansions had significantly higher NMSS scores compared to tremor-associated pathogenic expansion carriers (adj. *P* < 0.017) (Table 3).

**Table 3:** Demographic and clinical features in carriers of pathogenic repeats, NOTCH2NLC and GIPC1 pathogenic expansions

	Tremor-associated carriers, *n* = 6	*NOTCH2NLC* carriers, *n* = 27	*GIPC1* carriers, *n* = 3	*P* (All)	Adj. *P* (tremor-associated versus *NOTCH2NLC*)	Adj. *P* (tremor-associated versus *GIPC1*)	Adj. *P* (*NOTCH2NLC* versus *GIPC1*)
Sex, male, *n* (%)	3 (50.0)	10 (37.0)	2 (66.7)	0.614	0.659	1.000	0.548
Age, years, mean (SD)	51.0 (10.1)	54.0 (14.5)	45.7 (28.0)	0.674	0.451	0.905	0.647
Age at onset, years, mean (SD)	34.8 (19.1)	41.6 (13.7)	44.3 (19.5)	0.789	0.538	0.714	0.845
Duration (SD)	16.2 (15.2)	12.4 (8.8)	7.7 (4.2)	0.603	0.982	0.548	0.315
Education, years, mean (SD)	10.6 (2.9)	10.3 (4.0)	9.0 (1.0)	0.712	0.960	0.571	0.467
Head, *n* (%)	2 (33.3)	10 (27.0)	1 (33.3)	1.000	1.000	1.000	1.000
Face, *n* (%)	1 (16.7)	13 (48.1)	0 (0.0)	0.168	0.209	1.000	0.238
Voice, *n* (%)	3 (50.0)	10 (37.0)	0 (0.0)	0.437	0.659	0.464	0.532
Upper limbs, *n* (%)	6 (100.0）	27 (100.0)	3 (100.0)	NA	NA	NA	NA
Lower limbs, *n* (%)	3 (50.0)	12 (48.1)	0 (0.0)	0.439	1.000	0.464	0.238
Postural tremor, *n* (%)	6 (100.0）	27 (100.0)	3 (100.0)	NA	NA	NA	NA
Kinetic tremor, *n* (%)	6 (100.0）	24 (88.9)	3 (100.0)	NA	1.000	NA	1.000
Intention tremor, *n* (%)	4 (66.7)	14 (51.9)	3 (100.0)	0.271	0.665	0.500	0.238
TETRAS-I (SD)	16.3 (11.4)	27.1 (11.9)	9.3 (7.2)	**0.037**	0.109	0.393	0.026
TETRAS-II (SD)	19.0 (9.7)	29.3 (12.0)	13.0 (7.2)	0.060	0.122	0.393	0.061
MMSE (SD)	27.7 (2.1)	25.7 (3.0)	21.3 (10.7)	0.398	0.201	0.393	1.000
NMSS (SD)	5.2 (5.3)	23.4 (19.9)	10.0 (2.6)	**0.003**	**0.001**	0.262	0.072
ET-plus, *n* (%)	1 (16.7)	18 (66.7)	0 (0.0)	**0.013**	0.062	1.000	0.054
Dystonia, *n* (%)	0 (0.0)	4 (14.8)	0 (0.0)	1.000	1.000	NA	1.000
MCI, *n* (%)	0 (0.0)	2 (7.4)	3 (0.0)	1.000	1.000	NA	1.000
Tandem repeat, *n* (%)	0 (0.0)	5 (18.5)	0 (0.0)	0.721	0.556	NA	1.000
Rest tremor, *n* (%)	1 (16.7)	9 (33.3)	0 (0.0)	0.555	0.640	1.000	0.534

Adj. *P* = adjusted *P* (statistically significant cut-off of *P* < 0.017 by Bonferroni correction). Bold *P* value indicates statistical significance.

IA, intermediate allele; SD, standard deviation; ET, essential tremor; TETRAS, Tremor Research Group Essential Tremor Rating Assessment Scale; MMSE, Mini-Mental State Examination; NMSS, Non-Motor Symptoms Scale; MCI, mild cognitive impairment; NA, data not available.

### The phenotype of pathogenic expansion carriers

The main clinical manifestations observed in the six patients with pathogenic expansions were kinetic and postural tremors affecting both upper limbs, with an average score of 16.8 ± 12.7 for TETRAS-I and 19.8 ± 10.6 for TETRAS-II (Tables 2 and 4). None of the four patients carrying SCAs pathogenic expansion showed prominent cerebellar ataxia signs, parkinsonism, pyramidal signs, dystonia or cognitive impairment. None of the six individuals with pathogenic (premutation) expansions responded positively to β-blockers (propranolol or arotinolol) treatment.

**Table 4:** Clinical features of patients with pathogenic tremor-associated repeat expansions

Patient ID	P-1	P-2	P-3	P-4	P-5	P-6
Pathogenic repeat expansions	*ATXN1* (43 repeats)	*ATXN2* (36 repeats)	*ATXN8OS* (188 repeats)	*PPP2R2B* (59 repeats)	*FMR1* (56 repeats)	*SAMD12* (105 repeats)
Sex	Male	Male	Male	Female	Female	Male
Age (years)	37	49	57	53	66	44
Age at onset (years)	7	46	17	49	55	35
Duration (years)	30	3	40	4	11	9
Education (years)	11	9	11	6	15	12
Tremor distribution						
Upper limbs	+	+	+	+	+	+
Head	−	−	+	+	−	−
Face	−	−	−	+	−	−
Voice	−	−	+	+	−	+
Lower limbs	−	+	+	−	+	−
Tremor type						
Kinetic tremor	+	+	+	+	+	+
Postural tremor	+	+	+	+	+	+
Rest tremor	−	+	−	−	−	−
Intention tremor	−	−	+	+	+	+
TETRAS-I	14	1	27	32	9	15
TETRAS-II	15	5.5	26.5	33	19.5	14.5
MMSE	28	24	27	30	28	29
NMSS	1	2	9	14	4	1
Ataxia/pyramidal signs/parkinsonism/muscle cramps/limb weakness/dysphagia/dysarthria/cognition impairment	−	−	−	−	−	−
Response to β-blockers	−	−	−	−	−	−
MRI cerebellar atrophy	−	Mild increase in the width of cerebellar folia and sulci	−	−	−	−

TETRAS, Tremor Research Group Essential Tremor Rating Assessment Scale; MMSE, Mini-Mental State Examination; NMSS, Non-Motor Symptoms Scale.

**Figure 2:**
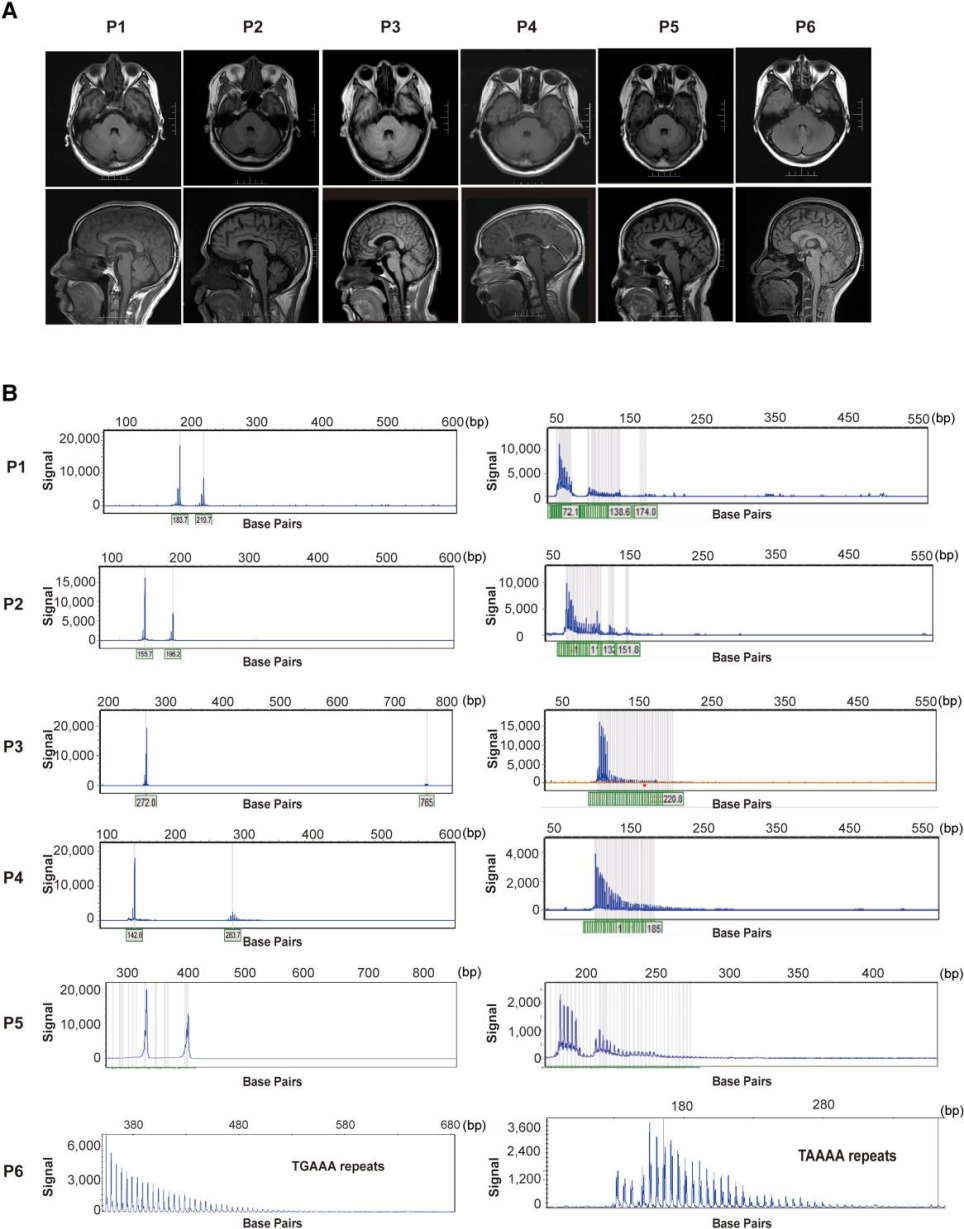
**MRI and electropherograms of pathogenic expansion carriers.** (**A**) The brain MRI of patients carried six patients with pathological expansions. (**B**) Electropherograms of patients with repeat expansions of tremor-associated STRs. STRs, short tandem repeats.

Patient P-1 is a 37-year-old male, carrying 43 repeats in the *ATXN1* gene, with tremor onset at a very young age. Despite the worsening amplitude and frequency of tremors over time, pyramidal or cerebellar signs were not observed. Patient P-2 is a 49-year-old male, carrying 36 repeats in the *ATXN2* gene. While these SCAs expanded carriers did not exhibit significant abnormalities in cerebellar MRI, P-2 displayed a slight enlargement of cerebellar folia and sulci (Fig. 2A). Patient P-3 is a 57-year-old female, carrying 188 repeats in the *ATXN8OS* gene. Tremors involve the voice and upper limbs, and mild tremor was observed in the lower limbs. Cognitive impairment was not found in this patient. Patient P-4 is a 53-year-old female, carrying 59 repeats in the *PPP2R2B* gene. The tremor spread beyond the upper extremities, leading to severe tremors affecting the head, voice, face and lower limbs during the first year of disease duration.

Patient P-5 is a 66-year-old female, carrying 56 GGC repeats in the *FMR1* gene and has been experiencing upper limb tremors for eleven years with no signs of ataxia, cognitive impairment or autonomic dysfunction. Brain MRI did not reveal any notable atrophy or white matter disease.

Patient P-6 is a 44-year-old female, carrying an insertion of 105 TTTCA repeats in the *SAMD12* gene (Fig. 2B). The brain MRI did not reveal any apparent abnormalities. Notably, electromyography displayed short irregular bursts indicative of a cortical origin, which is a typical feature in FCMTE. Although the proband has never experienced seizures, his mother had two episodes of epileptic seizures during her youth.

## Discussion

In this study, we performed analyses of repeat expansion size in tremor-associated STRs in a large familial ET cohort. In this study, we identified 12 (2.5%) patients carrying intermediate repeat expansions and 6 (1.2%) with pathogenic expansions. Our findings suggest that patients primarily diagnosed with ET may carry pathogenic expansions in known tremor-associated STRs. Of interest is whether these repeat expansions display pleiotropy, or if they have been misdiagnosed.

Intermediate STR expansions typically do not directly cause the disease associated with a pathogenic expansion at the locus. However, they can contribute to or increase the risk of developing related diseases and phenotypes. For instance, both *ATXN1* and *ATXN2* intermediate expansions are associated with an increased risk of developing amyotrophic lateral sclerosis.^32^  *HTT* intermediate expansions give rise to the risk of multisystem atrophy,^33^ and could confer late-onset abnormal motor and cognitive phenotype.^34^ Our results suggest that intermediate and pathogenic repeat expansions of these tremor-associated STRs did not increase the risk of familial ET or influence phenotypes of ET patients.

We identified six patients carrying pathogenic expansions, each with a distinct gene involved: *ATXN1*, *ATXN2*, *ATXN8OS*, *PPP2R2B*, *FMR1* and *SAMD12*. The misdiagnosis rate of ET has been reported to range from 37% to 50%.^35,36^ It is plausible that patients carrying tremor-associated STRs were coincidentally misdiagnosed due to an ET-like phenotype, and they might have developed classic symptoms associated with SCAs, FXTAS or FCMTE if they had lived longer. This highlighted the significance of recognizing the potential overlap and underscores the necessity for follow-up assessments of patients presenting with an ET phenotype. No significant differences were observed in demographics and clinical features between carriers of pathogenic expansions and non-carriers. Notably, previous studies have revealed an association between *NOTCH2NLC* expansion and ET,^5,28^ and it exhibited a relatively high prevalence (4.6%) in our familial ET cohort. When compared to patients carrying *SCAs*, *FMR1* and *SAMD12* pathogenic expansion, those with *GIPC1* expansion did not show significant differences. However, *NOTCH2NLC* expansions exhibited more severe non-motor symptoms and daily life impairment, and demonstrated a tendency to have a higher prevalence of ET-plus. It seems that patients carrying *NOTCH2NLC* with more complex symptoms, which may be related to more complex and diffuse central and peripheral neurodegeneration.^37^ Previous study indicated that a percentage of these patients display symptoms related to neuronal intranuclear inclusion disease during follow-up.^28^

To gain deeper insights, we meticulously reviewed the medical history and clinical features of each patient with pathogenic expansions, aiming to identify any distinguishing characteristics from those typically observed in ET. Limited literature suggested a prevalence of 0.5% (1/177) *ATXN3* in ET.^38^ However, pathogenic or intermediate expansions were not reported in 10 common degenerative hereditary ataxias among 323 ET patients in the study conducted by Louis *et al*.,^39^ and neither Nicoletti *et al*.^40^ nor Chen *et al*. ^41^ found *PPP2R2B* in ET patients. Previous studies were limited to a small ET population or lack of consideration of family history. In our study, we observed a prevalence of 0.8% (4/515) for SCAs (*ATXN1*, *ATXN2*, *ATXN8OS* and *PPP2R2B*). These patients with SCA expansions exhibited typical upper limbs tremors, without significant signs of cerebellar dysfunction. SCA1 is characterized by progressive cerebellar ataxia, and affected individuals may have gait disturbances and slurred speech at an early stage of disease. Patient P-1 carried 43 repeats of the *ATXN1* gene and showed tremors in the upper limbs as the sole symptom for the duration of 30 years without developing ataxia. Previous reports have indicated that alleles with 36–44 CAG repeats interrupted by CATs in the *ATXN1* gene are typically non-pathogenic.^42,43^ The RP-PCR results of P-1 showed an interruption, and the expansion in *ATXN1* was probably not the real pathogenic cause in this case. P-2 showed a mild increase in the width of cerebellar folia and sulci in brain MRI, which may indicate early cerebellar changes. P-3 carried 188 repeats of the *ATXN8OS* gene and suffered tremors for over 40 years but showed no impaired tandem gait or other ataxia signs. SCA8 typically presents as very slowly progressive ataxia, and several unusual non-ataxia phenotypes have been reported, including amyotrophic lateral sclerosis, Parkinson's disease, seizure-like episodes with migraine, etc.^44,45^ ET may be also one of the mimic phenotypes of SCA8. The underlying mechanism remains uncovered. Tremor has been reported in SCA1 (5.8%), SCA2 (27.5%), SCA8 (42.9%) and are most common in SCA12 (90%),^46,47^ and tremor in SCA12 often leading to misdiagnosis as ET.^48^ We observed prominent tremors of extremities, lip, head and trunk in the second year of the duration of P-4, and tremors progress without sign of ataxia in the fourth year of disease. SCA12 is also a slowly progressive disorder, and it is challenging to determine whether P-4 is ET or exhibiting early symptoms of SCA12 based on the current symptoms, and further follow-up of the patient is necessary for a conclusive diagnosis. The severity of expansion-related diseases is generally correlated with the size of the expansion range. It is noteworthy that except for SCA8, most of the pathogenic genes identified in ET showed a relatively low end of the pathogenic range (Fig. 1), which may lead to patients in our study showing tremors as mild symptoms other than typical symptoms of SCAs.

FCMTE presents with tremor-like cortical myoclonus, which can mimic ET.^49,50^ The electrophysiologic investigations have provided substantial evidence supporting a cortical origin with giant somatosensory evoked potentials, an enhanced C reflex at rest and pre-myoclonus cortical spikes by jerk locked averaging method (JIA).^51^ In the case of P-5, the patient predominantly displayed kinetic and postural tremors of upper limbs, with no history of seizures. Notably, the patient's mother reported having experienced two episodes of seizure history. The inclusion of a family history of neurological diseases and the utilization of electrophysiological assessments can provide valuable assistance in the clinical diagnosis of FCMTE.

Patients with FXTAS may initially exhibit action tremor or intention tremor as the sole clinical symptom in the early stages. Previous research has reported a prevalence of grey zone in 0.0–1.5% of ET patients and premutation in 2.7% of patients with presumed ET and additional neurological features.^52-54^ In our study, we identified ‘grey zone’ repeats in 0.4% (2/515) of ET patients, and identified in a female ET patient with premutation. Patient P-6, a 66-year-old female carried 56 repeats, which falls within the lower range of the premutation threshold. This patient did not show any cognitive impairment, and brain MRI was normal. It has been reported that some patients presented with a predominant phenotype of kinetic tremor without cognitive impairment or specific MRI features of FXTAS, indicating that the possible existence of background genetic modifiers may influence the phenotype.^55^

Tremors are prevalent in approximately half of the 37 known neurological disorders associated with STR expansions. These tremors tend to emerge early in the course of these disorders, resulting in phenotypic overlap with ET, and other symptoms may occur with disease progression. Therefore, our research highlights the importance of considering these STR mutations in familial ET, and ET patients should undergo long-term follow-up. It seems the patients who carry low-range pathogenic expansions of tremor-associated STRs are more likely to exhibit symptoms resembling those of ET. Additionally, the interrupt motif or genetic modifiers may also contribute to the clinical characteristics.

## Conclusions

Our study identified tremor-associated STRs intermediate and pathogenic expansions in a large familial ET cohort. Intermediate repeat expansions of these STRs were not associated with an increased risk for these patients, and rare pathogenic expansion in theses STRs did not significantly impact the general clinical features. Diagnosis of ET based solely on clinical symptoms remains challenging, necessitating long-term follow-ups, auxiliary diagnosis and a detailed survey of family history in familial ET patients.

## Supplementary Material

fcae217_Supplementary_Data

## Data Availability

The data are not publicly available due to privacy or ethical restrictions. The data are available upon reasonable request after ethics clearance and approval from the corresponding authors.
