# Supplementary material for: Tremor-associated short tandem repeat intermediate and pathogenic expansions in familial essential tremor
Source: Brain Commun. 2024 Jun 29;6(4):fcae217. doi: 10.1093/braincomms/fcae217 (PMC11220504; doi:10.1093/braincomms/fcae217)
Supplement: fcae217_Supplementary_Data [file fcae217_supplementary_data.pdf]

Supplementary Table 1. The ranges of expanded repeats in tremor-associated short tandem repeats.

Gene (Disease)	Repeat sequence	Intermediate range	Pathogenic range	Reference
ATXN1 (SCA1)	CAG	36-41	41-81	[1]
ATXN2 (SCA2)	CAG	32-33	>34	[2]
ATXN3 (SCA3)	CAG	45-60	60-87	[3]
CACNA1A (SCA6)	CAG	19-20	20-33	[4]
ATXN7 (SCA7)	CAG	28-35	35->200	[5]
ATXN8OS (SCA8)	CTG	51-70	70-1300	[6]
ATXN10 (SCA10)	ATTCT	22-850	850-4500	[7]
PPP2R2B (SCA12)	CAG	43-50	50-78	[8]
TBP (SCA17)	CAG	41-48	49	[9]
BEAN1 (SCA31)	TGGAA insertion		>110	[10]
NOP56 (SCA36)	GGCCTG	15-650	650-2500	[11]
DAB1 (SCA37)	ATTTC insertion		31-75	[12]
ATN1 (DRPLA)	CAG	35-49	49-88	[13]
FMR1 (FXTAS)	GGC	45-54*	55-200*	[14]
SAMD12 （FCMTE1）	TTTCA insertion			[15]

*The intermediate range of *FMR1* gene is 45-54, and the premutation range of *FMR1* gene is 55-200.

Supplementary Figure 1. Correlation between age at onset and repeat size of tremor-associated STRs in 515 familial ET probands. (BEAN1 (SCA31), DAB1 (SCA37) and SAMD12 (FCMTE1) are not shown since the disease is caused by alternative pathogenic repeat insertions). Covariance analysis was performed to assess the relationship between repeat size and age at onset. The R^2 and P value was shown in figure. STRs, short tandem repeats.

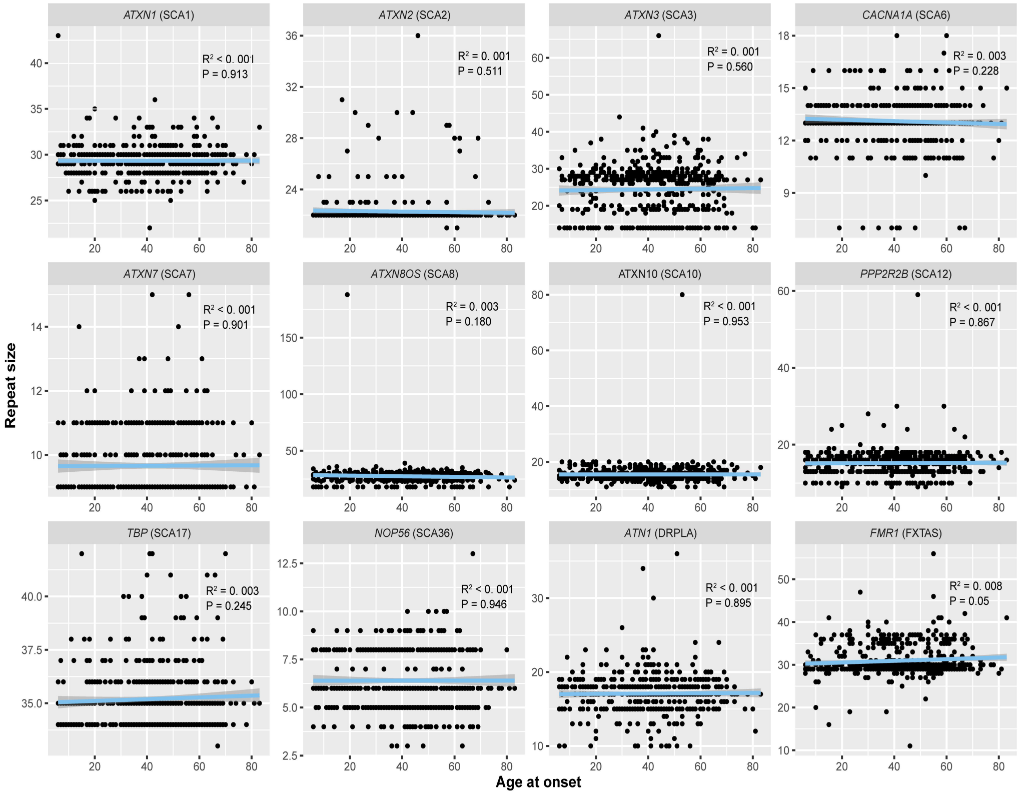

Supplementary Table 2. Demographic and clinical features in carriers of *SCAs* , *FMR1* and *SAMD12* expansion carriers.

	SCAs expansion carriers, n = 14	*FMR1* expansion carriers, n = 3	*SAMD12* expansion carriers, n=1	Non-carriers, n = 467	Adj. P (*SCAs* vs Non-carriers)	Adj. P (*FMR1* vs Non-carriers)	Adj. P (*SAMD12* vs Non-carriers*)
Sex, male, n (%)	7 (50.0)	2 (66.7)	Male	254 (54.4)	0.745	1.000	NA
Age, years, mean (SD)	55.4 (14.7)	53.0 (21.7)	44	53.8 (16.1)	0.715	0.915	NA
Age at onset, years, mean (SD)	42.8 (18.8)	45.7 (16.2)	35	41.8 (16.7)	0.715	0.657	NA
Duration (SD)	12.6 (10.7)	7.3 (5.5)	9	11.9 (9.6)	0.883	0.412	NA
Education, years, mean (SD)	10.7 (3.7)	18.0 (4.2)	12	10.2 (4.0)	0.885	0.046	NA
Head tremor, n (%)	4 (28.6)	0 (0.0)	0	153 (32.8)	0.968	0.554	NA
Face tremor, n (%)	3 (21.4)	0 (0.0)	0	122 (26.1)	0.932	0.572	NA
Voice tremor, n (%)	4 (28.6)	0 (0.0)	1	131 (28.1)	1.000	0.563	NA
Upper limbs tremor , n (%)	14 (100.0)	3 (100.0)	1	467 (100.0)	NA	NA	NA
Lower limbs tremor, n (%)	4 (28.6)	2 (66.7)	0	134 (28.7)	1.000	0.202	NA
Postural tremor, n (%)	14 (100.0)	3 (100.0)	1	467 (97.9)	1.000	1.000	NA
Kinetic tremor, n (%)	14 (100.0)	3 (100.0)	1	453 (97.0)	1.000	1.000	NA
Intention tremor, n (%)	6 (42.9)	2 (66.7)	1	178 (38.1)	0.783	0.561	NA
TETRAS-I , mean (SD)	13.4 (11.5)	7.5 (2.1)	15	13.4 (9.7)	0.846	0.366	NA
TETRAS-II , mean (SD)	19.7 (8.5)	16.8 (3.9)	14.5	17.4 (7.8)	0.355	0.922	NA
MMSE , mean (SD)	28.1 (2.1)	28.5 (0.7)	29	27.7 (2.8)	0.752	0.964	NA
NMSS, mean (SD)	8.4 (8.2)	2.0 (2.8)	1	12.1 (15.5)	0.750	0.177	NA
ET-plus, n (%)	4 (28.6)	2 (66.7)	0	234 (50.1)	0.288	0.585	NA
Dystonia, n (%)	0 (0.0)	1 (33.3)	0	37 (7.9)	0.557	0.224	NA
MCI, n (%)	1 (7.1)	1 (33.3)	0	107 (22.9)	0.164	0.554	NA

Tandem repeat, n (%)	13 (92.9)	0 (0.0)	0	54 (11.6)	0.609	1.000	NA
Rest tremor, n (%)	2 (14.3)	0 (0.0)	0	57 (12.2)	1.000	1.000	NA

Abbreviation: IA, intermediate allele; SD, standard deviation; ET, essential tremor; TETRAS, Tremor Research Group Essential Tremor Rating Assessment Scale; MMSE, Mini-Mental State Examination NMSS, Non-Motor Symptoms Scale, MCI, Mild cognitive impairment; NA, data not available.

*Comparison analysis was not performed in *SAMD12* because of the limitation in number

Adj. P = adjusted P (statistically significant cutoff of $P < 0.025$ by Bonferroni correction).

Supplementary References

1. Zühlke C, Dalski A, Hellenbroich Y, Bubel S, Schwinger E, Bürk K. Spinocerebellar ataxia type 1 (SCA1): phenotype-genotype correlation studies in intermediate alleles. *European journal of human genetics : EJHG*. Mar 2002;10(3):204-9. doi:10.1038/sj.ejhg.5200788
2. Daoud H, Belzil V, Martins S, et al. Association of long ATXN2 CAG repeat sizes with increased risk of amyotrophic lateral sclerosis. *Archives of neurology*. Jun 2011;68(6):739-42. doi:10.1001/archneurol.2011.111
3. Toulis V, Casaroli-Marano R, Camós-Carreras A, et al. Altered retinal structure and function in Spinocerebellar ataxia type 3. *Neurobiol Dis*. Aug 2022;170:105774. doi:10.1016/j.nbd.2022.105774
4. Wiethoff S, O'Connor E, Haridy NA, et al. Sequencing analysis of the SCA6 CAG expansion excludes an influence of repeat interruptions on disease onset. *Journal of neurology, neurosurgery, and psychiatry*. Nov 2018;89(11):1226-1227. doi:10.1136/jnnp-2017-317253
5. Stevanin G, Giunti P, Belal GD, et al. De novo expansion of intermediate alleles in spinocerebellar ataxia 7. *Hum Mol Genet*. Oct 1998;7(11):1809-13. doi:10.1093/hmg/7.11.1809
6. Cleary JD, Subramony SH, Ranum LPW. Spinocerebellar Ataxia Type 8. In: Adam MP, Feldman J, Mirzaa GM, et al, eds. *GeneReviews(®)*. University of Washington, Seattle
Copyright © 1993-2024, University of Washington, Seattle. GeneReviews is a registered trademark of the University of Washington, Seattle. All rights reserved.; 1993.
7. Matsuura T, Ashizawa T. Spinocerebellar Ataxia Type 10. In: Adam MP, Feldman J, Mirzaa GM, et al, eds. *GeneReviews(®)*. University of Washington, Seattle
Copyright © 1993-2024, University of Washington, Seattle. GeneReviews is a registered trademark of the University of Washington, Seattle. All rights reserved.; 1993.
8. Srivastava AK, Takkar A, Garg A, Faruq M. Clinical behaviour of spinocerebellar ataxia type 12 and intermediate length abnormal CAG repeats in PPP2R2B. *Brain : a journal of neurology*. Jan 2017;140(1):27-36. doi:10.1093/brain/aww269
9. Toyoshima Y, Onodera O, Yamada M, Tsuji S, Takahashi H. Spinocerebellar Ataxia Type 17. In: Adam MP, Feldman J, Mirzaa GM, et al, eds. *GeneReviews(®)*. University of Washington, Seattle
Copyright © 1993-2024, University of Washington, Seattle. GeneReviews is a registered trademark of the University of Washington, Seattle. All rights reserved.; 1993.
10. Sato N, Amino T, Kobayashi K, et al. Spinocerebellar ataxia type 31 is associated with "inserted" penta-nucleotide repeats containing (TGGAA)n. *American journal of human genetics*. Nov 2009;85(5):544-57. doi:10.1016/j.ajhg.2009.09.019
11. García-Murias M, Quintáns B, Arias M, et al. 'Costa da Morte' ataxia is spinocerebellar ataxia 36: clinical and genetic characterization. *Brain : a journal of neurology*. May 2012;135(Pt 5):1423-35. doi:10.1093/brain/aws069
12. Seixas AI, Loureiro JR, Costa C, et al. A Pentanucleotide ATTTC Repeat Insertion in the Non-coding Region of DAB1, Mapping to SCA37, Causes Spinocerebellar Ataxia. *American journal of human genetics*. Jul 6 2017;101(1):87-103. doi:10.1016/j.ajhg.2017.06.007
13. Nowak B, Kozlowska E, Pawlik W, Fiszer A. Atrophin-1 Function and Dysfunction in Dentatorubral-Pallidoluysian Atrophy. *Movement disorders : official journal of the Movement Disorder Society*. Apr 2023;38(4):526-536. doi:10.1002/mds.29355
14. Tassone F, Iong KP, Tong TH, et al. FMR1 CGG allele size and prevalence ascertained through newborn screening in the United States. *Genome Med*. 2012;4(12):100. doi:10.1186/gm401
15. Cen Z, Jiang Z, Chen Y, et al. Intronic pentanucleotide TTTCA repeat insertion in the SAMD12

gene causes familial cortical myoclonic tremor with epilepsy type 1. *Brain : a journal of neurology*. Aug 1 2018;141(8):2280-2288. doi:10.1093/brain/awy160
